# Long Noncoding RNA NRAV Promotes Respiratory Syncytial Virus Replication by Targeting the MicroRNA miR-509-3p/Rab5c Axis To Regulate Vesicle Transportation

**DOI:** 10.1128/JVI.00113-20

**Published:** 2020-05-04

**Authors:** Jian Li, Miao Li, Xiuli Wang, Mengfei Sun, Cuiqing Ma, Wenzhang Liang, Xue Gao, Lin Wei

**Affiliations:** a Department of Immunology, Hebei Medical University, Shijiazhuang, Hebei, China; b Key Laboratory of Immune Mechanism and Intervention on Serious Disease in Hebei Province, Shijiazhuang, Hebei, China; c Department of Pathogen Biology, Hebei Medical University, Shijiazhuang, Hebei, China; d The Second Hospital of Hebei Medical University, Shijiazhuang, Hebei, China; Hudson Institute of Medical Research

**Keywords:** NRAV, Rab5c, respiratory epithelial cells, long noncoding RNA, miR-509-3p, respiratory syncytial virus

## Abstract

Respiratory syncytial virus (RSV) is an enveloped RNA virus which is responsible for approximately 80% of lower respiratory tract infections in children. Current lines of evidence have supported the functional involvement of long noncoding RNA (lncRNA) in many viral infectious diseases. However, the overall biological effect and clinical role of lncRNAs in RSV infection remain unclear. In this study, lncRNAs related to respiratory virus infection were obtained from the lncRNA database, and we collected 144 clinical sputum specimens to identify lncRNAs related to RSV infection. Quantitative PCR (qPCR) detection indicated that the expression of lncRNA negative regulator of antiviral response (NRAV) in RSV-positive patients was significantly lower than that in uninfected patients, but lncRNA psoriasis-associated non-protein coding RNA induced by stress (PRINS), nuclear paraspeckle assembly transcript 1 (NEAT1), and Nettoie Salmonella pas Theiler’s (NeST) showed no difference *in vivo* and *in vitro*. Meanwhile, overexpression of NRAV promoted RSV proliferation in A549 and BEAS-2B cells, and vice versa, indicating that the downregulation of NRAV was part of the host antiviral defense. RNA fluorescent *in situ* hybridization (FISH) confirmed that NRAV was mainly located in the cytoplasm. Through RNA sequencing, we found that Rab5c, which is a vesicle transporting protein, showed the same change trend as NRAV. Subsequent investigation revealed that NRAV was able to favor RSV production indirectly by sponging microRNA miR-509-3p so as to release Rab5c and facilitate vesicle transportation. The study provides a new insight into virus-host interaction through noncoding RNA, which may contribute to exploring potential antivirus targets for respiratory virus.

**IMPORTANCE** The mechanism of interaction between RSV and host noncoding RNAs is not fully understood. In this study, we found that the expression of long noncoding RNA (lncRNA) negative regulator of antiviral response (NRAV) was reduced in RSV-infected patients, and overexpression of NRAV facilitated RSV production *in vitro*, suggesting that the reduction of NRAV in RSV infection was part of the host antiviral response. We also found that NRAV competed with vesicle protein Rab5c for microRNA miR509-3p in cytoplasm to promote RSV vesicle transport and accelerate RSV proliferation, thereby improving our understanding of the pathogenic mechanism of RSV infection.

## Abstract (precis)

The mechanism of interaction between RSV and host noncoding RNAs is not fully understood. In this study, we found that the expression of long noncoding RNA (lncRNA) negative regulator of antiviral response (NRAV) was reduced in RSV-infected patients, and overexpression of NRAV facilitated RSV production *in vitro*, suggesting that the reduction of NRAV in RSV infection was part of the host antiviral response. We also found that NRAV competed with vesicle protein Rab5c for microRNA miR509-3p in cytoplasm to promote RSV vesicle transport and accelerate RSV proliferation, thereby improving our understanding of the pathogenic mechanism of RSV infection.

## INTRODUCTION

Respiratory syncytial virus (RSV), an enveloped single-negative-strand RNA virus (1), is one of the main pathogens causing acute respiratory infection (2) and asthma (3). Globally, RSV infection accounts for approximately 3.2 million hospitalizations annually among children younger than 5 years (4). Despite years of research, the mechanism of interaction between RSV and host cells has not been fully elucidated, so much so that RSV infection is still prevalent, and a side effect-free vaccine has not been successfully developed (5).

Transcriptome studies and genomic microarrays have shown that more than 90% of genomes are transcribed to noncoding RNAs (6). Depending on its distinctive long transcript, secondary structure, and subcellular location (7), long noncoding RNA (lncRNA) can regulate gene expression at different levels (8). Numerous reports have shown that lncRNAs play important roles in the process of viral infection (9, 10). For example, following human immunodeficiency virus (HIV) infection, late protein Vpu induces host cells to express lncRNA noncoding repressor of nuclear factor of activated T cells (NFAT) (NRON) at a high level; afterward, NRON binds to the NFAT scaffold complex, resulting in transcriptional inhibition of HIV (11). Hepatitis C virus (HCV) can upregulate the expression of lncRNA Lethe, and Lethe inhibits the transcriptional activity of NF-κB, leading to the promotion of HCV replication (12).

As one of the leading pathogens in the respiratory tract, RSV will inevitably cause changes in the expression of some noncoding RNAs in host cells during infection. Here, we explored the probable host lncRNAs involved in RSV infection. Based on the results from clinical samples and a subsequent RNA-seq assay, we focused on lncRNA negative regulator of antiviral response (NRAV) and revealed the cross talk among NRAV, microRNA miR-509-3p, and Rab5c in cytoplasm, shedding new light on the regulation of noncoding RNA in RSV infection.

## RESULTS

### NRAV was downregulated in RSV-infected patients and mainly located in the cytoplasm.

**FIG 1:**
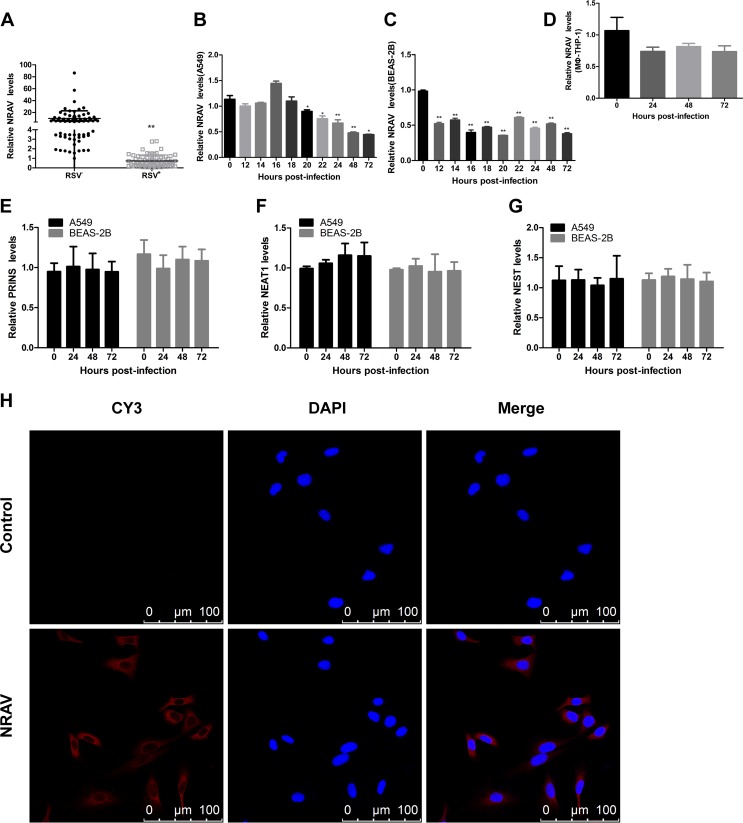
Human NRAV was downregulated by RSV infection. (A) The expression of NRAV in 144 clinical samples was analyzed by qRT-PCR (RSV^–^ 75, RSV^+^ 69). (B to D) A549, BEAS-2B, or MΦ-THP-1 cells were infected with RSV (MOI = 3) for the indicated number of hours. Relative NRAV levels were determined by qRT-PCR. (E to G) A549 or BEAS-2B cells were infected with RSV for the indicated number of hours (MOI = 3). Relative PRINS (E), NEAT1 (F), and NeST (G) levels were determined by qRT-PCR. (H) The localization of NRAV in A549 was examined by RNA-FISH (scale bar = 100 μm). Data are shown as mean ± SD for three independent experiments. *, *P* < 0.05; **, *P* < 0.01.

To identify host lncRNAs that were influenced by RSV infection, we retrieved four lncRNAs associated with RNA virus or respiratory virus infection in lncRNAdb database, i.e., NEAT1 (GenBank accession number NC_000011.10), PRINS (GenBank accession number HG975433.1), NeST (GenBank accession number NR_104124.1), and NRAV (GenBank accession number NR_038854). The relative expression levels of these lncRNAs were determined by reverse transcription-quantitative PCR (qRT-PCR) in 144 clinical sputum specimens (RSV^–^, *n* = 75, RSV^+^, *n* = 69) (Table 1). The results showed that NRAV expression in the RSV-infected group was lower than that in the uninfected groups (Fig. 1A), while NEAT1, PRINS, and NeST were not detected in these sputum samples.

**TABLE 1:** Demographic and clinical information

Characteristic	No. of cases (%)
Specimens	144
RSV^+^	69 (47.92)
RSV^–^	75 (52.08)
Season	
19 September 2018–31 December 2018	91 (63.19)
1 January 2019–25 February 2019	53 (36.81)
Location	
Inpatient	144 (100)
Outpatient	0
ED^*a*^	0
Age, yrs	
Range (yrs)	2–13
RSV^+^ <6	48 (33.33)
RSV^+^ ≥6	21 (14.58)
RSV^–^ <6	39 (27.08)
RSV^–^ ≥6	36 (25.00)
Sex	
Male	66 (45.83)
Female	78 (54.17)

a ED, emergency department.

We then observed lncRNA expression levels *in vitro*. Regarding the different cell types in sputum, Guiot reported that there were macrophages (49%), squamous or epithelial cells (23%), lymphocytes (3%), eosinophils (1%) and so on, which could be divided into immune cells and epithelial cells (13). Considering that the proportion of lymphocytes and eosinophils in sputum is much lower than other cell types, we selected macrophages and epithelial cells to evaluate NRAV levels. For epithelial cells, A549 or BEAS-2B cells were infected with RSV for 72 h. Total RNA was collected at indicated time points since infection, and qRT-PCR was performed. NRAV was reduced in A549 (Fig. 1B) or BEAS-2B (Fig. 1C) cells during RSV infection, but PRINS, NEAT1, and NeST showed no difference (Fig. 1E to G). Similarly, for macrophages, the MΦ-THP-1cells (14) were infected and detected in the same manner. However, the expression of NRAV was decreased slightly in MΦ-THP-1cells, and the difference was not statistically significant (Fig. 1D). As a result, NRAV was significantly downregulated in epithelial cells after RSV infection, and we mainly focused on A549 and BEAS-2B *in vitro* for the following study.

Subsequently, we explored the conservation of NRAV in the LNCipedia database (https://lncipedia.org/db/transcript/NRAV:3), and NRAV shared no locus conservation with mouse. In addition, RNA FISH was performed in A549 to determine the subcellular location of NRAV. Data showed that NRAV was located in both the cytoplasm and nucleus but mainly in the cytoplasm (Fig. 1H).

### NRAV promoted RSV replication *in vitro*.

**FIG 2:**
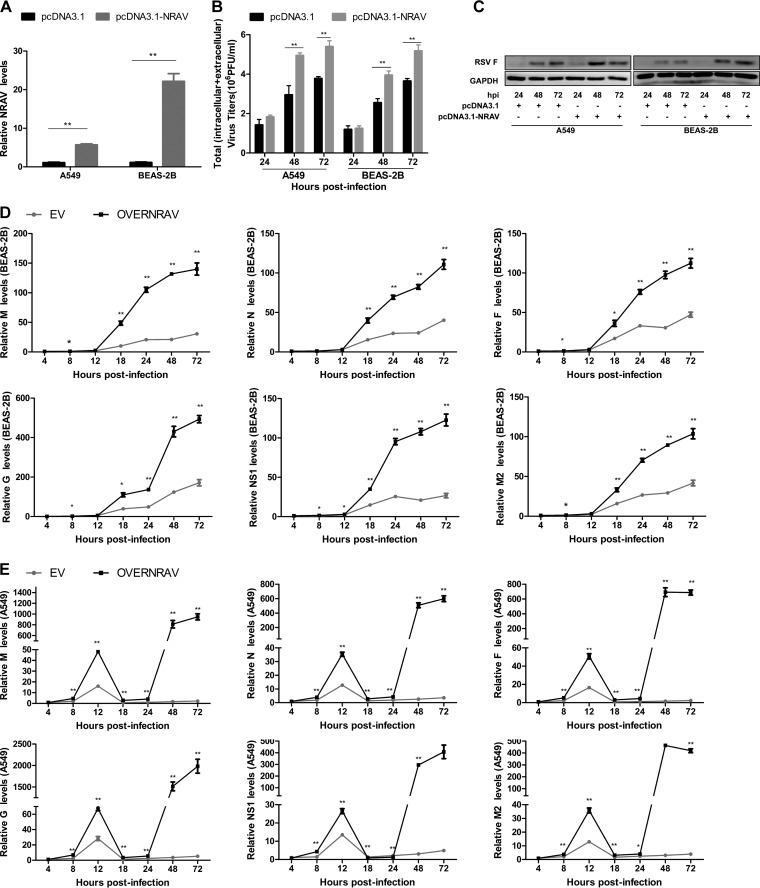
Overexpression of NRAV promoted RSV replication. (A) The overexpression of NRAV was analyzed by RT-qPCR in A549 and BEAS-2B cells. (B) RSV replication was examined by plaque assay in NRAV overexpressed A549 or BEAS-2B cells infected with RSV. Virus titers were measured at different hours postinfection. (C) Expression of RSV F protein was measured at the indicated time by immunoblotting in NRAV overexpressed A549 or BEAS-2B cells infected with RSV. (D and E) Expression of RSV genes was analyzed at the indicated time by RT-qPCR in NRAV overexpressed BEAS-2B (D) or A549 (E) cells infected with RSV. Data are shown as mean ± SD for three independent experiments. *, *P* < 0.05; **, *P* < 0.01.

The full length of the NRAV sequence has already been reported (15). To evaluate the influence of NRAV on RSV proliferation, we constructed pcDNA3.1-NRAV containing the whole length of NRAV to express NRAV ectopically *in vitro*, and qRT-PCR was performed to determine NRAV expression (Fig. 2A). Both NRAV-overexpressed cells and the control group were infected by RSV (multiplicity of infection [MOI] = 3), and cell lysates were harvested at indicated time points to evaluate RSV replication by virus plaque assay (Fig. 2B), immunoblotting (Fig. 2C), and qRT-PCR (Fig. 2D and E). Results implied that overexpression of NRAV induced higher RSV titer, more F protein expression, and higher RSV gene levels.

### Knockdown of NRAV weakened RSV proliferation *in vitro*.

**FIG 3:**
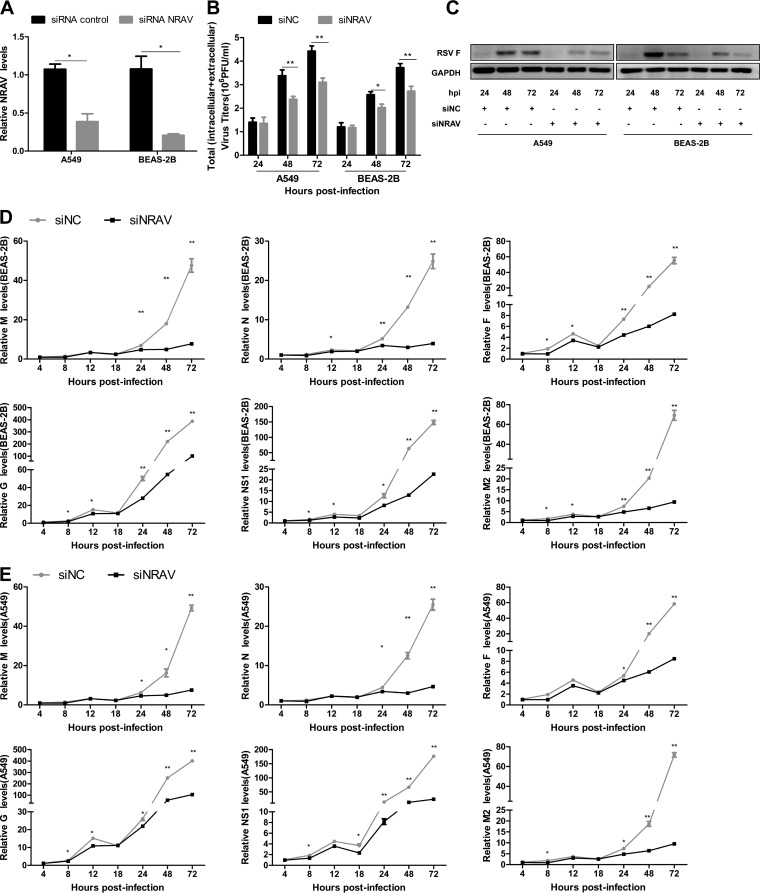
Knocking down NRAV inhibited RSV replication. (A) siRNA-based knockdown of NRAV was analyzed by RT-qPCR in A549 and BEAS-2B cells. (B) RSV replication was examined by plaque assay in NRAV knocked-down cells infected with RSV. Virus titers were measured at different hours postinfection. (C) Expression of RSV F protein was measured at the indicated time by immunoblotting in NRAV knocked-down A549 or BEAS-2B cells infected with RSV. (D and E) Expression of RSV genes was analyzed at the indicated time by RT-qPCR in NRAV knocked-down BEAS-2B (D) or A549 (E) cells infected with RSV. Data are shown as mean ± SD for three independent experiments. *, *P* < 0.05; **, *P* < 0.01.

To further validate the functionality of NRAV on RSV proliferation, we generated NRAV-specific small interfering RNA (siRNA) to silence NRAV ectopically *in vitro*, and qRT-PCR was performed to determine NRAV expression (Fig. 3A). Both NRAV knocked-down cells and the control group were infected by RSV (MOI = 3). Cell lysates were harvested at indicated time points to analyze RSV proliferation by virus plaque assay (Fig. 3B), immunoblotting (Fig. 3C), and qRT-PCR (Fig. 3D and E). The results showed that downregulation of NRAV induced lower RSV titer, less F protein expression, and lower RSV gene levels.

### NRAV was positively correlated with Rab5c and was a direct target of miR-509-3p.

**FIG 4:**
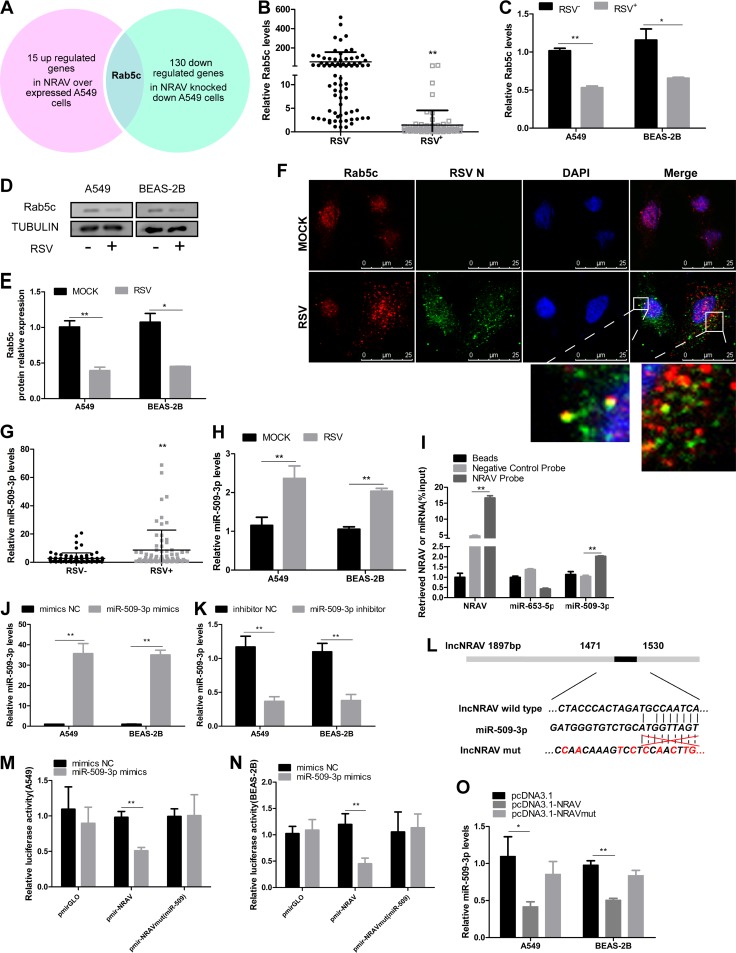
NRAV was positively correlated with Rab5c and interacted with miR-509-3p. (A) RNA sequence analysis revealed 15 upregulated genes in NRAV overexpressed A549 cells (*n* = 3) compared to the controls (*n* = 3) and 130 downregulated genes in NRAV knocked-down A549 cells (*n* = 1) compared to the control (*n* = 1) (*P* < 0.05; fold change, ≥ 2). There was only one molecule, Rab5c, at the overlap of the two groups. (B) The expression of Rab5c in clinical samples was analyzed by qRT-PCR (RSV^–^ 75, RSV^+^ 69). (C) A549 or BEAS-2B cells were infected with RSV (MOI = 3) for 48 h. Relative Rab5c levels were determined by qRT-PCR. (D and E) Expression of Rab5c protein was measured by immunoblotting in cells infected with or without RSV for 48 h. Rab5c protein levels were quantitated by densitometry and normalized to Tubulin (E). (F) The subcellular localization of Rab5c (red) was examined by immunofluorescence (scale bar = 25 μm) in relation to RSV N protein (blue) in A549 cells infected with RSV (MOI = 3) for 1 h (lower panel) or mock-infected cells (upper panel). (G) The expression of miR-509-3p in clinical samples was analyzed by qRT-PCR (RSV^–^ 75, RSV^+^ 69). (H) A549 or BEAS-2B cells were infected with RSV (MOI = 3) for 48 h. Relative miR-509-3p levels were determined by qRT-PCR. (I) A549 cell lysates were incubated with biotin-labeled NRAV; after pulldown, NRAV, miR-509-3p, and miR-653-5p were extracted and assessed by qRT-PCR. (J and K) The overexpression (J) or knockdown (K) of miR-509-3p in A549 and BEAS-2B cells were analyzed by RT-qPCR. (L) Predicted binding sites for miR-509-3p on NRAV wild or mutation type. (M and N) Luciferase activities were detected in A549 (M) or BEAS-2B (N) cells cotransfected with miR-509-3p and luciferase reporters containing nothing, NRAV, or mutant transcript. Data are presented as the relative ratio of firefly luciferase activity to renilla luciferase activity. (O) MiR-509-3p expression levels were analyzed by RT-qPCR after the transfection of pcDNA3.1-NRAV or NRAVmut into cells. Data are shown as mean ± SD for three independent experiments. *, *P* < 0.05; **, *P* < 0.01.

To investigate which mRNAs were affected by NRAV expression, genome-wide mRNA sequencing was performed in cells as described below. In the overexpression group, pcDNA3.1-NRAV was transfected into A549 cells (*n* = 3), while pcDNA3.1 empty vector was transfected as the control (*n* = 3). In the knockdown group, NRAV siRNA (si-NRAV) (*n* = 1) and negative-control siRNA (si-control) (*n* = 1) were transfected into A549 cells. Data implied that there were 15 upregulated mRNAs in NRAV-overexpressed cells compared with the controls (see Table S1 in the supplemental material), and a total of 130 downregulated mRNAs were found in NRAV knocked-down cells (Table S2) (*P* < 0.05; fold change, ≥2). Interestingly, in the overlap of the two comparisons, we found only one molecule, Rab5c (GenBank accession number NM_201434.3), which was in the same trend as NRAV (Fig. 4A). To further confirm the mRNA sequence result, Rab5c levels were determined by qRT-PCR in clinical sputum specimens (RSV^–^ 75, RSV^+^ 69), which were the same samples used in Fig. 1A, and Rab5c expression in RSV-infected samples was lower than that in uninfected ones (Fig. 4B). Moreover, we examined Rab5c expression by qRT-PCR (Fig. 4C) or immunoblotting (Fig. 4D and E) *in vitro*, and RSV infection induced lower Rab5c expression than in the controls. The above results indicated that Rab5c was positively correlated with NRAV during RSV infection.

As an early endosomal marker protein, Rab5 is mainly involved in cellular vesicle transport and endocytosis (16, 17). The Rab5 family contains Rab5a, b, and c, which share similar function and location (18, 19). It has been reported that RSV entered respiratory epithelial cells in the form of macropinocytosis, which was then transformed by a Rab5-positive macropinosome. The RSV expression level was decreased significantly when the cells were transfected with Rab5 dominant negative mutant (20), suggesting that the Rab5 molecule was directly involved in the intracellular transmission of RSV virus. Furthermore, we performed an immunofluorescence assay, and confocal microscopy showed that RSV could be colocalized with Rab5-positive vacuoles in the cytoplasm (Fig. 4F), which was consistent with Krzyzaniak’s report (20).

The function of lncRNA is closely related to its subcellular location (7). Based to the results showing that NRAV was mainly located in the cytoplasm and influenced Rab5c expression in the same trend, we speculated that Rab5c might be the target molecule of NRAV in respiratory epithelial cells.

Numerous cytoplasmic lncRNAs have been reported to perform as competing endogenous RNAs (ceRNAs) by binding common microRNAs (miRNAs) (21). We analyzed the target miRNA of NRAV and Rab5c via bioinformatics (lncrnadb and RegRNA 2.0). The results suggested that both NRAV and Rab5c had the potential to bind miR-509-3p; that is, they might be connected by the same miRNA response element (miR-509-3p) in cytoplasm. What’s more, miR-509-3p had been reported to target the 3′ UTR of Rab5c (22).

Additionally, we evaluated the expression of miR-509-3p in clinical sputum specimens (Fig. 4G) which were the same samples used in Fig. 1A, and the expression of miR-509-3p was further examined in A549 and BEAS-2B cells (Fig. 4H). Data showed that infection of RSV induced higher expression of miR-509-3p *in vivo* and *in vitro*. To investigate the direct association between NRAV and miR-509-3p, we generated a biotin-labeled NRAV probe and a negative-control (NC) probe. A549 cell lysates were incubated with the probes overnight. The specific binding was confirmed by affinity pulldown of endogenic miR-509-3p and miR-653-5p (used as a control). MiR-509-3p and miR-653-5p were then extracted and assessed by qRT-PCR. Meanwhile, the NRAV level was examined by qRT-PCR to evaluate the efficacy of NRAV pulldown (Fig. 4I). Data showed that endogenic miR-509-3p, other than miR-653-5p, could be pulled down by biotin-labeled NRAV.

To further validate the binding, miR-509-3p mimics and inhibitor were generated and verified by qRT-PCR *in vitro* (Fig. 4J and K). Luciferase reporters containing NRAV (1,471 to 1,530 bp) wild-type or mutated miR-509-3p binding sites were constructed (Fig. 4L) and cotransfected with miR-509-3p mimics or the mimic control. It was indicated that overexpression of miR-509-3p weakened the luciferase activities of wild-type pmirGLO-NRAV but not those of mutant type or empty vector (Fig. 4M and N). We constructed pcDNA3.1-NRAVmut containing full-length wild-type NRAV with point mutations in miR-509-3p binding sites to express NRAVmut ectopically. Expression of miR-509-3p was decreased in the pcDNA3.1-NRAV (wild-type) group but not in empty vector or mutant vectors (Fig. 4O).

The results implied that NRAV was positively correlated with Rab5c *in vivo* and *in vitro*, and NRAV was physically associated with miR-509-3p.

### NRAV/miR-509-3p association regulated RSV proliferation.

**FIG 5:**
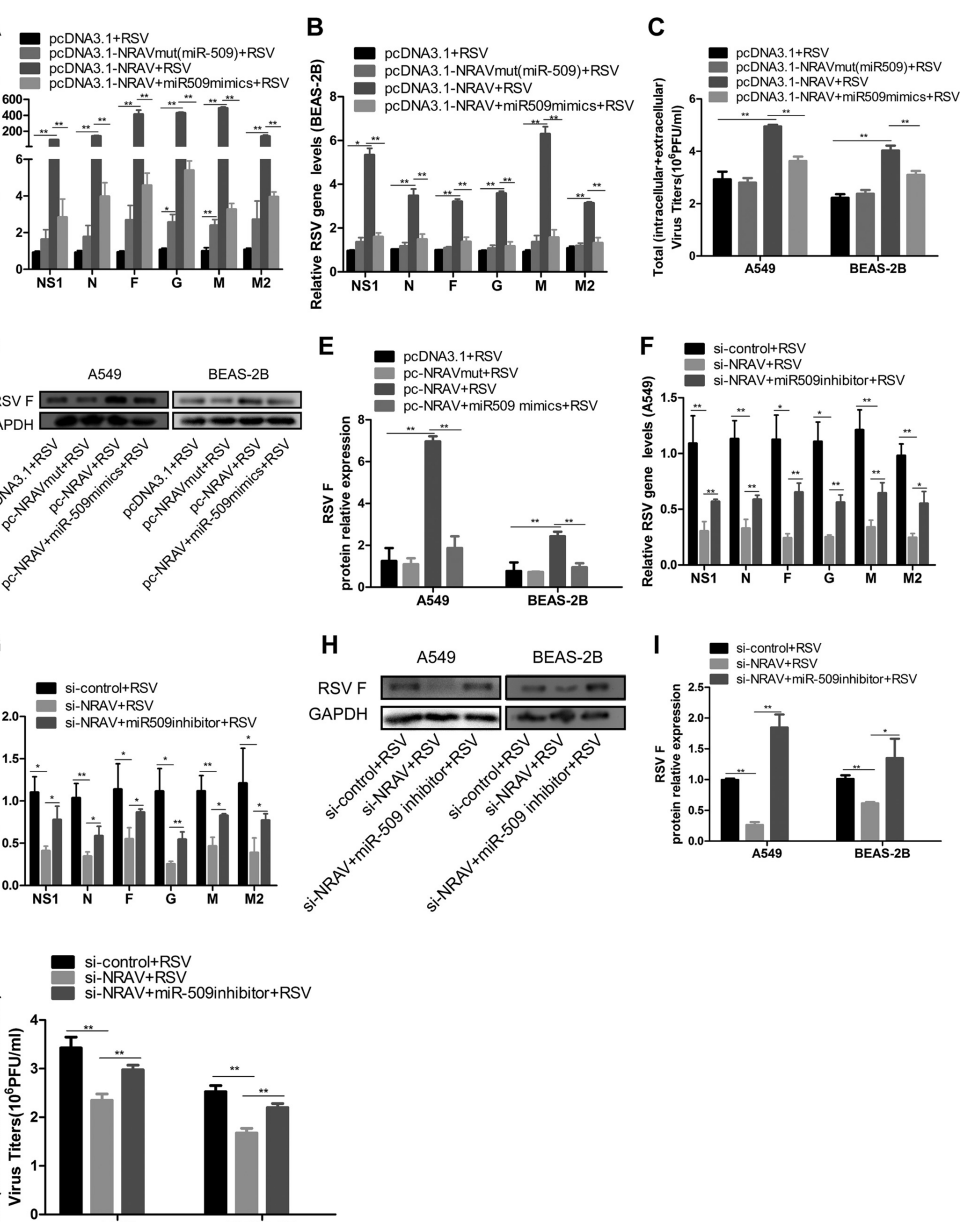
NRAV/miR-509 association regulated RSV expression. (A and B) Expression of RSV genes was analyzed by qRT-PCR in A549 (A) or BEAS-2B (B) cells transfected with pcDNA3.1-NRAV or pcDNA3.1-NRAVmut or cotransfected with pcDNA3.1-NRAV and miR-509-3p mimics. (C) RSV replication was examined by plaque assay in A549 or BEAS-2B cells transfected with pcDNA3.1-NRAV or pcDNA3.1-NRAVmut or cotransfected with pcDNA3.1-NRAV and miR-509-3p mimics. (D and E) Expression of RSV F protein was measured by immunoblotting in A549 or BEAS-2B cells transfected with pcDNA3.1-NRAV or pcDNA3.1-NRAVmut or cotransfected with pcDNA3.1-NRAV and miR-509-3p mimics (D). RSV F expression levels were quantitated by densitometry and normalized to GAPDH (E). (F and G) Expression of RSV genes was analyzed by qRT-PCR in A549 (F) or BEAS-2B (G) cells cotransfected with si-NRAV and miR-509-3p inhibitor. (H and I) Expression of RSV F protein was measured by immunoblotting in cells cotransfected with si-NRAV and miR-509-3p inhibitor (H). RSV F expression levels were quantitated by densitometry and normalized to GAPDH (I). (J) RSV replication was examined by plaque assay in cells cotransfected with si-NRAV and miR-509-3p inhibitor. Data are shown as mean ± SD for three independent experiments. *, *P* < 0.05; **, *P* < 0.01.

To explore whether NRAV regulated RSV proliferation via modulating miR-509-3p, we transfected cells with pcDNA3.1 empty vector, pcDNA3.1-NRAV, or pcDNA3.1-NRAVmut. In the rescue group, pcDNA3.1-NRAV and miR-509-3p mimics were cotransfected into cells. The next day, the cells listed above were infected with RSV (MOI = 3) for 48 h. The cell lysates were harvested to evaluate RSV replication by qRT-PCR, virus plaque assay, and immunoblotting. Results indicated that overexpression of NRAV, but not NRAVmut, increased RSV production. Furthermore, miR-509-3p mimics rescued the enhancement in NRAV overexpressed cells in RSV gene levels (Fig. 5A and B), virus titer (Fig. 5C), and F protein expression (Fig. 5D and E).

On the other hand, in the silencing experiment, cells were transfected with si-control or si-NRAV. In the rescue group, si-NRAV and miR-509 inhibitor were cotransfected into cells. From the next day on, the cells were infected by RSV (MOI = 3) for 48 h. Data suggested that silencing NRAV reduced RSV production, and miR-509-3p inhibitor abolished this decrease in RSV gene expression (Fig. 5F and G), F protein level (Fig. 5H and I), and viral titer (Fig. 5J). Accordingly, the results revealed that miR-509-3p was able to reverse the effect of NRAV on RSV replication through miR-509-3p/NRAV interaction.

### NRAV regulated Rab5c expression via sponging miR-509-3p.

**FIG 6:**
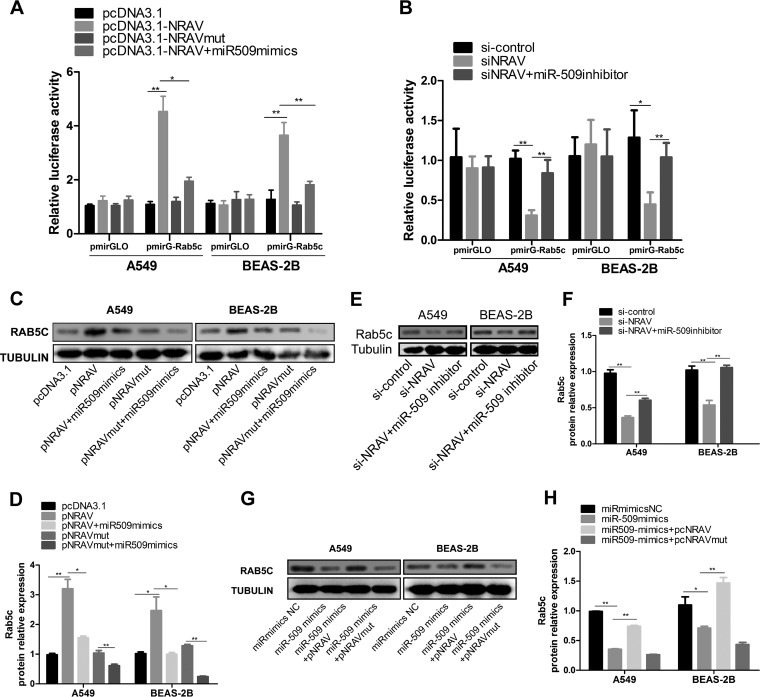
NRAV/miR-509 association regulated Rab5c expression. (A and B) Luciferase activities were evaluated in A549 or BEAS-2B cells transfected with luciferase reporters containing Rab5c 3′ UTR or nothing. Data are presented as the relative ratio of firefly to renilla luciferase activity. (C and D) Expression of Rab5c protein was measured by immunoblot analysis in A549 or BEAS-2B cells transfected with pcDNA3.1-NRAV or pcDNA3.1-NRAVmut or cotransfected with miR-509-3p mimics (C). Rab5c expression levels were quantitated by densitometry and normalized to tubulin (D). (E and F) Expression of Rab5c protein was measured by immunoblot analysis in A549 or BEAS-2B cells cotransfected with si-NRAV and miR-509-3p inhibitor (E). Rab5c expression levels were quantitated by densitometry and normalized to tubulin (F). (G and H) Expression of Rab5c protein was measured by immunoblot analysis in A549 or BEAS-2B cells transfected with miR-509-3p mimics or cotransfected with pcDNA3.1-NRAV or pcDNA3.1-NRAVmut (G). Rab5c expression levels were quantitated by densitometry and normalized to tubulin (H). Data are shown as mean ± SD for three independent experiments. *, *P* < 0.05; **, *P* < 0.01.

We further identified whether NRAV regulated Rab5c expression through binding miR-509-3p. Luciferase plasmid pmirGLO-Rab5c was constructed containing the 3′ UTR of Rab5c. PmirGLO-Rab5c or empty vector was transfected into cells. A dual luciferase reporter assay suggested that overexpression of NRAV, but not NRAVmut, enhanced luciferase activity of pmirGLO-Rab5c. In the rescue experiment, miR-509-3p mimics abrogated high luciferase activities in NRAV overexpressed cells (Fig. 6A). On the other hand, knocking down of NRAV reduced luciferase activity of pmirGLO-Rab5c. Reciprocally, miR-509-3p inhibitor rescued low luciferase activities in NRAV knocked-down cells (Fig. 6B).

Subsequently, we examined such a relationship through immunoblotting. Overexpression of NRAV, but not NRAVmut, increased Rab5c expression, and miR-509-3p mimics reversed the high Rab5c expression in NRAV overexpressed cells. In NRAVmut overexpressed cells, miR-509-3p mimics made Rab5c expression lower than the empty control group (Fig. 6C and D). Knocking down of NRAV weakened Rab5c expression, which was abolished by miR-509-3p inhibitor (Fig. 6E and F). Meanwhile, miR-509-3p mimics reduced Rab5c protein levels *in vitro*, and overexpression of NRAV, but not NRAVmut, reversed this decrease (Fig. 6G to H). The results suggested that NRAV was positively associated with Rab5c via competitive binding of miR-509-3p in respiratory epithelial cells, which was called the NRAV/miR-509-3p/Rab5c axis.

### The effect of NRAV on RSV was realized by Rab5c.

**FIG 7:**
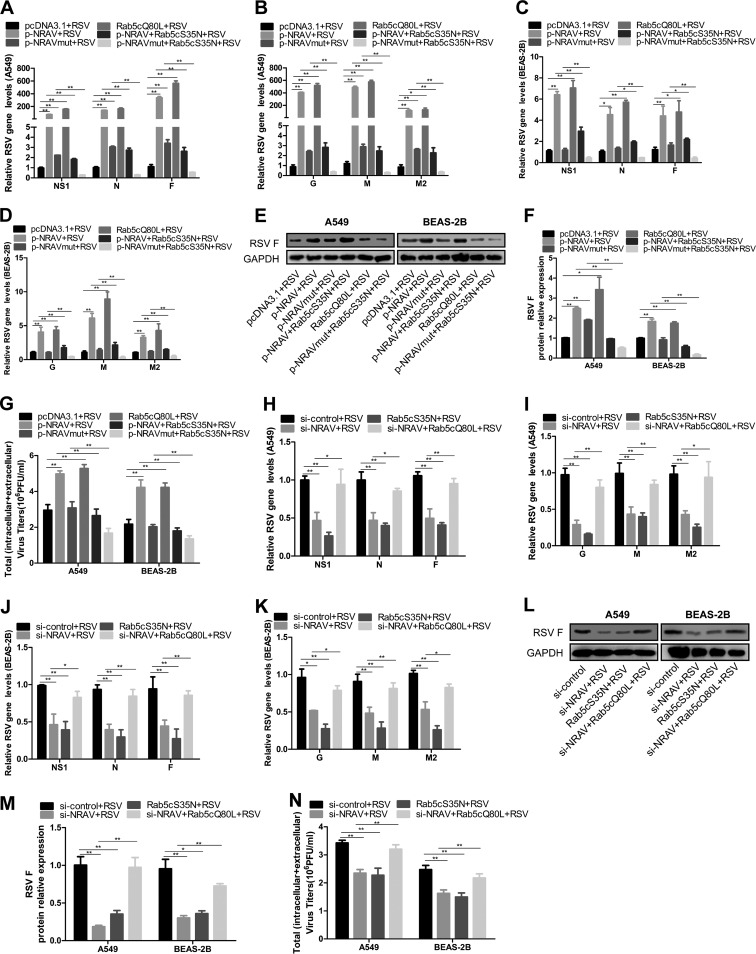
NRAV regulated RSV infection by Rab5c. (A to D) Expression of RSV genes was analyzed by qRT-PCR in A549 (A and B) or BEAS-2B (C and D) cells transfected with pcDNA3.1-NRAV, pcDNA3.1-NRAVmut, or Rab5cQ80L or cotransfected with Rab5cS35N. (E and F) Expression of RSV F protein was measured by immunoblot analysis in cells transfected with pcDNA3.1-NRAV, pcDNA3.1-NRAVmut, or Rab5cQ80L or cotransfected with Rab5cS35N (E). RSV F expression levels were quantitated by densitometry and normalized to GAPDH (F). (G) RSV replication was examined by plaque assay in cells transfected with pcDNA3.1-NRAV, pcDNA3.1-NRAVmut, or Rab5cQ80L or cotransfected with Rab5cS35N. (H to K) Expression of RSV genes was analyzed by qRT-PCR in A549 (H and I) or BEAS-2B (J and K) cells transfected with si-NRAV or Rab5cS35N or cotransfected with Rab5cQ80L. (L and M) Expression of RSV F protein was measured by immunoblot analysis in cells transfected with si-NRAV or Rab5cS35N or cotransfected with Rab5cQ80L (L). RSV F expression levels were quantitated by densitometry and normalized to GAPDH (M). (N) RSV replication was examined by plaque assay in cells transfected with si-NRAV or Rab5cS35N or cotransfected with Rab5cQ80L. Data are shown as mean ± SD for three independent experiments. *, *P* < 0.05; **, *P* < 0.01.

To verify the role of Rab5c in the NRAV/miR-509-3p/Rab5c axis during RSV infection, we constructed a constitutive active (C/A) mutant and a dominant negative (D/N) mutant of Rab5c, i.e., Rab5cQ80L and Rab5cS35N (23). A549 or BEAS-2B cells were transfected by appointed plasmids, and from the next day on, the cells listed above were infected by RSV (MOI = 3) for 48 h. Data showed that overexpression of both NRAV and Rab5cQ80L increased RSV gene levels (Fig. 7A to D), F protein expression (Fig. 7E and F), and virus titer (Fig. 7G). Moreover, Rab5cS35N reversed such rises in NRAV overexpressed cells. In NRAVmut overexpressed cells, Rab5cS35N made RSV expression lower than the empty control group (Fig. 7A to G).

In addition, both si-NRAV and Rab5cS35N reduced RSV gene expression (Fig. 7H to K), F protein level (Fig. 7L and M), and viral titer (Fig. 7N), and Rab5cQ80L rescued these reductions in NRAV knocked-down cells (Fig. 7H to N).

**FIG 8:**
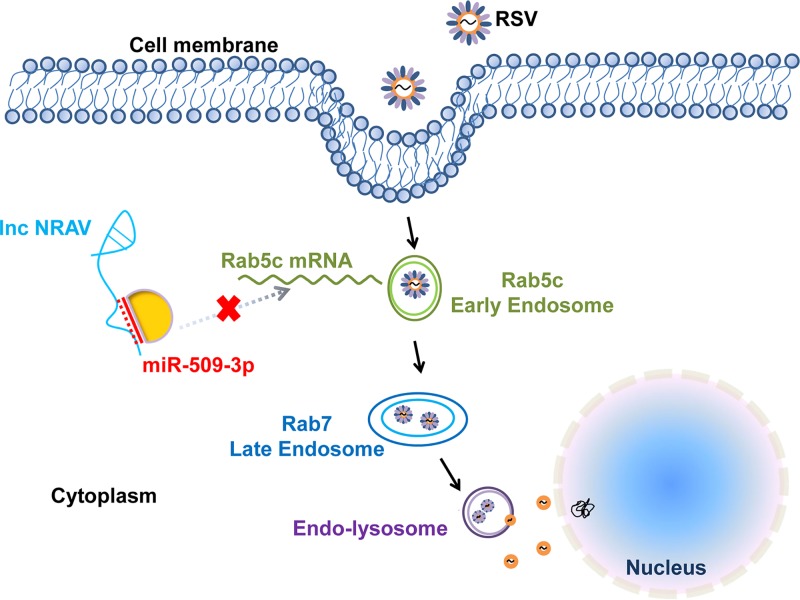
A schematic model of the NRAV/miR-509/Rab5c axis in RSV infection.

All the results revealed that NRAV influenced the expression of RSV via the NRAV/miR-509-3p/Rab5c axis so that it regulated the RSV infection process through Rab5c-positive vesicle transport (Fig. 8).

## DISCUSSION

In this study, NRAV was downregulated in RSV-infected samples. In addition, NRAV promoted RSV proliferation by competitively binding miR-509-3p, enhancing expression of Rab5c, and accelerating intracellular vesicle transport of RSV.

Over the past decade, researchers have identified differentially expressed lncRNAs in diverse viral infections by whole-transcriptome sequencing and established several lncRNA databases (24), such as NONCODE (http://www.noncode.org) and lncRNAdb. Given that RSV is an RNA virus (25) that infects the respiratory tract (26), we have found four candidate lncRNAs which can be influenced by RNA virus or respiratory virus via bioinformatics retrieval, including NEAT1, PRINS, NeST, and NRAV. We designed and synthesized primers for subsequent validation.

In order to confirm RSV infection, sputum specimens of pediatric inpatients were collected and detected by a multiple detection kit for 13 respiratory pathogens. The RNAs prepared from sputum specimens were randomly collected from 75 full-negative specimens and 69 RSV-single-positive specimens to verify bioinformatics results. Detected by qRT-PCR, however, only NRAV was differentially expressed in RSV-infected clinical samples and cell lines. Thus, we focused on NRAV for functional study. On the other hand, we will explore NRAV levels in patients infected with other respiratory viruses in a future study.

In this study, functional experiments were carried out *in vitro*, because NRAV had no conserved locus in mouse. Results indicated that RSV production could be improved when NRAV was overexpressed, and replication of RSV was inhibited when silencing NRAV. The experiment was carried out in A549 and BEAS-2B cells in functional experiments. The A549 cell line is derived from human adenocarcinoma type II alveolar cells, and BEAS-2B was developed from primary human bronchial epithelial cells transformed with adenovirus type 12 (Ad12)-SV40. Different characteristics were observed in these two cell lines during RSV infection. Hillyer (27) has reported that A549 cells are permissive to RSV infection and produce proinflammatory genes after RSV infection. Instead, BEAS-2B cells could restrict the infection and produce molecules characteristic of an antiviral reaction. Consequently, A549 cells were highly susceptible to RSV infection, whereas BEAS-2B cells limited infection to small individual foci or cells during RSV infection. Similar to those results, in our study, compared with BEAS-2B, when A549 cells were infected with RSV, the increase of RSV gene expression was much more obvious. Furthermore, overexpression of NRAV led to robust enhancement of RSV genes in A549, and the degree of increase was significantly higher than that in BEAS-2B cells (Fig. 5A and B).

During the pcDNA3.1-based overexpression or siRNA-based knockdown experiment, it was interesting that the expression features of RSV genes were quite different between the empty vector (EV) group (Fig. 2) and the si-control group (Fig. 3). It is widely acknowledged that transfection of an NC siRNA or empty vector DNA might have a negligible impact on cells. In effect, however, Stepanenko and Heng have reported that a range of molecular manipulations from the NC siRNA or empty vector will probably influence the biological system, resulting in types of nonspecific effects on cells in the NC group. Furthermore, during the transient transfection, due to differences in nucleic acid type, total base amount, and molecular structure, the nonspecific impact from empty vector backbone RNA (pcDNA3.1, 5,428 bp) and siRNA (double-strand short interfering RNA, 21 bp) could differ from each other (28), which might induce the expression features of RSV genes that were quite different in the EV and siNC groups.

In order to pinpoint the downstream target of NRAV, four groups of A549 cells were used for RNA-seq, including over-NRAV versus the empty vector group (*n* = 3) and si-NRAV versus the si-control group (*n* = 1). It was inferred that 15 mRNAs were upregulated in over-NRAV versus the empty vector group, and 130 mRNAs were downregulated in si-NRAV versus the si-control group. Fortunately, only Rab5c was in the intersection of the two comparisons. Subsequently, the result was validated *in vivo* and *in vitro*, suggesting that Rab5c was positively correlated with NRAV.

The subcellular localization of lncRNA is one of the leading determinants of its molecular function (29). Based on the results that NRAV was mainly located in the cytoplasm subcelluarly and showed the same trend with Rab5c, we tentatively put forward the idea that NRAV and Rab5c might share the same miRNA in cytoplasm, which is a major miRNA pool. In the light of bioinformatics analysis and a previous report, miR-509-3p has been reported to be able to bind Rab5c (22). Meanwhile, NRAV was predicted to bind to miR-509-3p’s seed region (Fig. 4L). Subsequently, we combined RNA pulldown (Fig. 4I) and dual luciferase reporter assays (Fig. 4M and N) to identify the NRAV/miR-509-3p association. In addition, we found that the effect of NRAV on RSV could be reversed by miR-509-3p.

The total RNA and protein in Fig. 5F to I were all extracted at 48 h post-RSV infection. It is interesting that RSV F mRNA expression in Fig. 5F and G were not consistent with their protein levels in Fig. 5H and I. In order to clarify this phenomenon, we separately extracted total RNA at 24, 36, and 48 h to detect the expressions of the F gene. The results showed that whether the RNA was extracted 24, 36, or 48 h postinfection, silencing NRAV reduced RSV F expression, and miR-509-3p inhibitor could rescue this decrease. With the extension of time, however, the expression of the F gene changed dynamically in the rescue group. When the cells were infected for 24 or 36 h, miR-509-3p inhibitor could significantly rescue the expression of F in mRNA level (data not shown). Nevertheless, at 48 h postinfection, the recovery of F expression was not as high as that at 24 or 36 h. Interestingly, in the rescue group, the mRNA level of the F gene at 24 h postinfection was somewhat consistent with F protein at 48 h after infection. As a result, when both RNA and protein were extracted at 48 h postinfection, the mRNA abundance of the F gene might not have a linear relationship with the expression of the F protein.

On the other hand, dual luciferase reporter assay (Fig. 6A and B) and immunoblotting (Fig. 6C to H) were performed to verify that Rab5c could be positively influenced by NRAV, and similarly, the influence could be abolished by miR-509-3p. However, both RSV and Rab5c levels have not completely returned to NC levels. It is probable that NRAV is able to regulate virus proliferation in multiple ways, for example, an NRAV/histone modification/interferon-stimulated gene (ISG) mechanism (15).

It has been reported that Rab proteins, such as Rab5 and Rab11, play a critical role in the entry, assembly, and excretion of RSV. Krzyzaniak inferred that host cell entry and intracellular transmission of RSV depended on Rab5-positive macropinosomes (20). Brock and Utley reported that Rab11 and Rab11FIPs were essential in RSV assembly and budding (30, 31). In our study, A549 cells were infected with RSV (MOI = 3) for 1 h, which was the average time for internalization (20). Confocal microscopy showed that some of the RSV was colocalized with Rab5c-positive vacuoles in the cytoplasm (Fig. 4F). These results indicated that through targeting the miR-509-3p/Rab5c axis, NRAV might promote RSV infection at the entry and intracellular transmission stage.

In our previous study, we found that RSV induced autophagy in host cells to facilitate RSV replication (32). Meanwhile, it had been reported that Rab5, which was indispensable for the recruitment of Rab7 to PI3P-positive autophagosomes, was one of the important factors to maintain unobstructed autophagy flow (33). Subsequently, we will further explore whether the effect of autophagy on RSV proliferation is mediated by the NRAV/miR-509-3p/Rab5c axis.

In order to explore whether NRAV/Rab5c was correlated with the progress and prognosis of RSV infection, we defined the length of hospital stay as an index to reflect the progress and prognosis of RSV infection. Based on the median expression of NRAV, we defined the RSV-infected patients as high NRAV group (*n* = 35) and low NRAV group (*n* = 34). Then, we compared NRAV level and the length of hospital stay, and no correlation was found. In addition, we observed no correlation between Rab5c expression and the length of hospital stay (data not shown). As a result, it is unclear whether this insignificance is due to the small number of RSV-positive cases in this study or if there is truly no prognostic difference. More patients should be included in our future study.

Overall, we found that RSV could decrease NRAV expression in patients’ sputum specimens. Overexpression of NRAV promoted RSV proliferation *in vitro* and vice versa. NRAV could act as a ceRNA in the NRAV/miR-509-3p/Rab5c axis during RSV infection, thus promoting RSV vesicle transport and accelerating RSV entry (Fig. 8), suggesting that the downregulation of NRAV in RSV infection was part of the host antiviral defense. The results may facilitate improvement in exploring a potential noncoding RNA target for diagnosis and treatment of respiratory virus infection.

## MATERIALS AND METHODS

### Patients.

This study was conducted in accordance with the principles of the Declaration of Helsinki. Between 19 September 2018, and 25 February 2019, sputum specimens of pediatric inpatients were randomly collected with a sputum aspirator based on National Clinical Laboratory Procedures. Specimens were detected using a multiple detection kit for 13 respiratory pathogens (Health Gene Tech, Ningbo, China) in the Second Hospital of Hebei Medical University. Total RNA was collected and qualified according to the kit; 13 common respiratory pathogens were detected in sputum samples by RT-PCR and capillary electrophoresis, including influenza A virus H1N1 and H3N2, parainfluenza viruses, human metapneumovirus, influenza B viruses, respiratory syncytial virus, coronavirus, rhinoviruses, bocaviruse, *Chlamydia*, Mycoplasma pneumoniae, and adenoviruses. Seventy-five samples were randomly selected as negative controls, and the detected results of thirteen pathogens in these samples were all negative. For the experimental group, 69 samples were randomly selected, in which, of the 13 pathogens, only RSV was positive. The patients’ information is shown in Table 1. Written informed consent was obtained from all parents or guardians.

### Cells and virus.

HEp-2 cells and RSV A strain Long were maintained as previously described (32). HEp-2 cells were utilized to amplify RSV (34). Human alveolar epithelial cells (A549), human bronchial epithelial cells (BEAS-2B), and human monocytic leukemia cell line (THP-1) were maintained in our laboratory and grown in RPMI 1640 supplemented with 10% (vol/vol) fetal bovine serum (FBS) (Gibco) and antibiotics (penicillin and streptomycin) (Solarbio) in a humidified 5% CO_2_ atmosphere at 37°C. Monocytes (THP-1) were treated with phorbol 12-myristate 13-acetate (PMA, 15 ng/ml; Multiscience) and were differentiated into macrophages MΦ-THP-1 after 12 h.

### Viral infection and virus titer assay.

A549 or BEAS-2B cells were washed and incubated with RSV at a certain multiplicity of infection (MOI) or mock-infected (with medium alone) for 2 h in serum-free RPMI 1640. The supernatant was aspirated after adsorption, and cells were cultured with RPMI 1640 supplemented with 2% (vol/vol) FBS for the indicated times.

Cell lysates were harvested at the indicated time points. RSV titers were detected by plaque-forming assay (35) and expressed in PFU/milliliter cell lysates.

### RNA extraction and reverse transcription quantitative PCR (qRT-PCR).

Total RNA was isolated with RNAiso Plus reagent (9101; TaKaRa). First-strand cDNA was generated with a PrimeScript RT reagent kit (047A; TaKaRa). Real-time PCR was performed in an ABI Prism 7500 sequence detection system (Applied Biosystems) using PowerUp SYBR green master mix (A25742; Applied Biosystems). ACTB was employed as an endogenous control for lncRNA and mRNA. The primers are shown in Table 2 and were synthesized by Invitrogen.

**TABLE 2:** Primers used for RT-qPCR and probes used in RNA-FISH

Primer/probe/siRNA	Sequence
NRAV sense	5′-GGAGTTGATGCCTCCGAACA-3′
NRAV antisense	5′-ATGACCGGAGCTGAAAGGTG-3′
NEAT1 sense	5′-TTGTTCCAGAGCCCATGAT-3′
NEAT1 antisense	5′-TGAAAACCTTTACCCCAGGA-3′
NEST sense	5′-AAACGCTGGAGGAGAAGTCA-3′
NEST antisense	5′-TTCTCCTCCAGCGTTTTA-3′
PRINS sense	5′-GGCCCAGTGAGAACTACGGAA-3′
PRINS antisense	5′-TCATCTGAGCTTGAGTTAATCGGC-3′
RSV M2 sense	5′-CATGAGCAAACTCCTCACTGAACT-3′
RSV M2 antisense	5′-TCTTGGGTGAATTTAGCTCTTCATT-3′
RSV F sense	5′-TAAGCAGCTCCGTTATCACATCTC-3′
RSV F antisense	5′-ATTGGATGCTGTACATTTAGTTTTGC-3′
RSV M sense	5′-ATGTGTAATGTGTCCTTGGATGA-3′
RSV M antisense	5′-TGATTTCACAGGGTGTGGTTACA-3′
RSV G sense	5′-CGGCAAACCACAAAGTCACA-3′
RSV G antisense	5′-TTCTTGATCTGGCTTGTTGCA-3′
RSV N sense	5′-AAGGGATTTTTGCAGGATTGTTT-3′
RSV N antisense	5′-CTCCCCACCGTAGCATTACTTG-3′
RSV NS1 sense	5’-CACAACAATGCCAGTGCTACAA-3′
RSV NS1 antisense	5′-TTAGACCATTAGGTTGAGAGCAATGT-3′
ACTB sense	5′-TGACGTGGACATCCGCAAAG-3′
ACTB antisense	5′-CTGGAAGGTGGACAGCGAGG-3′
miR-509-3p sense	5′-GATTCGCTTGATTGGTACGTCTGT-3′
miR-509-3p antisense	5′-TATGCTTGTTCACGACACCTTCAC-3′
miR-653-5p sense	5′-TTCCTTGCGGTGTTGAAACA-3′
miR-653-5p antisense	5′-TATGGTTGTAGACGACTCCTTGAC-3′
NRAV probe-1	5′-CCAAGAACCAATGTAGGGTC-3′
NRAV probe-2	5′-TTGTGGATGAGGTGAGGAGA-3′
NRAV probe-3	5′-TGGAAGCTTCCGGATTCAGA-3′
NRAV probe-4	5′-GAGAGTGTTGGTCTACTCAG-3′
Negative Control (NC) probe	5′-GTGTAACACGTCTATACGCCCA-3′
si-NRAV sense	5′-GGCAGGAGCUCCCUAAAUATT-3′
si-NRAV antisense	5′-UAUUUAGGGAGCUAAUGCCAG-3′
miR-509-3p mimics sense	5′-UGAUUGGUACGUCUGUGGGUAG-3′
miR-509-3p mimics antisense	5′-ACCCACAGACGUACCAAUCAUU-3′
miR-509-3p mimics NC sense	5′-UUCUCCGAACGUGUCACGUTT-3′
miR-509-3p mimics NC antisense	5′-ACGUGACACGUUCGGAGAATT-3′
miR-509-3p inhibitor	5′-CUACCCACAGACGUACCAAUCA-3′
miR-509-3p inhibitor NC	5′-CAGUACUUUUGUGUAGUACAA-3′

MiRNA was isolated with an miRNA isolation kit (DP501; Tiangen Biotech). MiRNA first-strand cDNA was generated with an miRNA first-strand cDNA synthesis kit (KR211; Tiangen Biotech). An miRNA qPCR detection kit (SYBR green) (FP411; Tiangen Biotech) was used for real-time PCR. Hsa-miR-509-3p (MIMAT0002881) primers were produced by Applied Biological Materials (MPH01751). U6 (GenBank accession number NR_004394) (CD201-0145; Tiangen Biotech) was employed as an endogenous control for miRNA.

### RNA fluorescent *in situ* hybridization.

NRAV (GenBank accession number NR_038854) probe mix and NC probe are shown in Table 2. All the probes were labeled with CY3 fluorescent dye. RNA fluorescent in situ hybridization (RNA-FISH) was performed using a fluorescent *in situ* hybridization kit (Gene Pharma, China) following the manufacturer’s instructions. Fluorescence detection was performed with a confocal laser-scanning microscope (Leica TCS SP5).

### Plasmids and small RNAs.

The full lengths of wild-type NRAV and NRAVmut with point mutations in miR-509-3p binding sites (Gene Pharma, Suzhou, China) were synthesized and subcloned into the BamHI and EcoRI sites, respectively, of the pcDNA3.1^+^ vector (Invitrogen), called pcDNA3.1-NRAV and pcDNA3.1-NRAVmut.

Wild-type NRAV (bp positions 1471 to 1530), NRAVmut (bp positions 1471 to 1530) with point mutations in the miR-509-3p binding sites (bp positions 1497 to 1517), and 3′ UTR of wild-type Rab5c mRNA (Gene Pharma, Suzhou, China) were subcloned into the SacI and XhoI sites of the pmirGLO vector (Promega), named pmirGLO-NRAV, pmirGLO-NRAVmut, and pmirGLO-Rab5c.

The sequences of si-NRAV, the miR-509-3p mimics and inhibitor, and the miRNA control are shown in Table 2.

The dominant negative mutant Rab5c (Rab5cS35N), containing a mutation at residue 35 from serine to asparagines, as well as the Rab5c constitutive active mutant Rab5cQ80L (Gene Pharma, Suzhou, China), which mutated residue 80 from glutamine to leucine, were constructed into the BamHI and EcoRI sites, respectively, of the pcDNA3.1^+^ vector (Invitrogen), named pcDNA3.1-Rab5cS35N and pcDNA3.1-Rab5cQ80L.

### Cell transfection.

Cells were seeded in six-well plates. Plasmids or miRNAs were transfected into cells with Lipofectamine 2000 (Invitrogen) following the manufacturer’s instructions.

### RNA-seq analysis.

Total RNA of A549 cells was isolated and employed for RNA-seq analysis. Construction of the cDNA library and sequencing were performed by Shanghai Biotechnology Corporation using an Illumina HiSeq 2000 system. High-quality reads were mapped with Homo sapiens genome hg38, using Hisat2 version 2.0.4. The expression level of each gene was standardized to fragments per kilobase of exon model per million mapped reads (FPKM) using StringTie version 1.3.0 and trimmed mean of M values (TMM) (*P* < 0.05, fold change, ≥ 2).

### RNA pulldown assay.

The NRAV probe mix and NC probe were the same as those used in RNA-FISH, and the probes were modified with 3′, 5′ biotins. RNA pulldown was performed using an RNA pulldown kit (Gene Pharma, Suzhou, China) following the manufacturer’s instructions. NRAV and miRNAs bound to the pulldown beads were examined using qRT-PCR. The primers of hsa-miR-509-3p (MIMAT0002881) and hsa-miR-653-5p (MIMAT0003328) are presented in Table 2.

### Dual luciferase reporter assay.

Cells were seeded in 96-well plates. Then, 24 h after transfection, Fluc and Rluc activities were measured using a Dual Glo luciferase assay system (E2920; Promega), and luminescence was measured with BioTek Synergy HT2 multiscan spectrum. Relative ratio = (well A1 firefly luminescence/Renilla luminescence)/(control firefly luminescence/Renilla luminescence).

### Immunostaining and microscopy.

A549 cells were seeded on coverslips and transiently transfected with pcDNA3.1-Rab5c with Lipofectamine 2000 (Invitrogen) for 24 h, followed by mock or RSV infection (MOI = 3) for another 1 h. The cells were fixed in 4% paraformaldehyde, permeabilized in 0.5% Triton X-100, and blocked in 10% normal goat serum (SL038; Solarbio) for 30 min. The cells were incubated with primary antibodies anti-Rab5c (ab199530, 1:500) and anti-RSV N (ab94806, 1:500) overnight at 4°C. Secondary antibodies included Alexa 594-labeled goat anti-rabbit (ab150080, 1:500) and Alexa 488-labeled goat anti-mouse (ab150113, 1:500). Coverslips were mounted on the glass slides with DAPI Flouromount-G (0100-20; SouthernBiotech) for confocal imaging (Leica TCS SP5).

### Western blot analysis.

Total cell lysates were prepared with a radio immune-precipitation assay (RIPA) in an ice bath for 30 min and supplemented with 1 mM phenylmethylsulfonyl fluoride (PMSF; BL507A; BioSharp) and phosphate inhibitors (P1260; Solarbio). The protein concentration was determined with a NanoDrop 2000c spectrophotometer (EW-83061-12; Thermo Scientific). A total of 30 μg proteins was separated by 12% sodium dodecyl sulfate polyacrylamide gel electrophoresis (SDS-PAGE), and the bands were transferred to a polyvinylidene fluoride (PVDF) membrane (IPVH00010; Millipore), which was blocked with 5% nonfat milk. After incubation with antibodies specific to RSV F (ab94968; Abcam), human Rab5c (A7342; ABclonal), Alpha-tubulin (ARH4207; Antibody Revolution), or GAPDH (AP0063; Bioworld), the blots were incubated with horseradish peroxidase (HRP)-labeled goat anti-mouse IgG (ASS1007; Abgent) or HRP-labeled goat anti-rabbit IgG (ASS1009; Abgent) and were detected with Western Lightning plus-ECL reagent (NEL104001EA; PerkinElmer) using a Synoptics Syngene bio-imaging instrument (R114075; Synoptics). GAPDH or alpha-tubulin was used as a loading control for immunoblotting.

### Statistical analysis.

Data from three independent experiments were expressed as the mean ± standard deviation (SD). SPSS version 16.0 software was used for statistical analyses. The comparisons were performed by using Student’s *t* test or one-way analysis of variance (ANOVA). *P* < 0.05 was considered to indicate a statistically significant difference.

### Data availability.

The RNA-seq data set is available in the Gene Expression Omnibus (GEO) and can be accessed via the GSE series number GSE145371.

## Supplementary Material

Supplemental file 1

Supplemental file 2
